# 3-(2-Chloro­anilino)isobenzofuran-1(3*H*)-one^1^

**DOI:** 10.1107/S160053680800771X

**Published:** 2008-03-29

**Authors:** Mustafa Odabaşoğlu, Orhan Büyükgüngör

**Affiliations:** a Department of Chemistry, Faculty of Arts and Sciences, Ondokuz Mayıs University, TR-55139 Kurupelit Samsun, Turkey; b Department of Physics, Faculty of Arts and Sciences, Ondokuz Mayıs University, TR-55139 Kurupelit Samsun, Turkey

## Abstract

In the mol­ecule of the title compound, C_14_H_10_ClNO_2_, the essentially planar phthalide group is oriented at a dihedral angle of 59.43 (4)° with respect to the substituted aromatic ring. In the crystal structure, inter­molecular C—H⋯O and N—H⋯O hydrogen bonds link the mol­ecules, generating *R*_4_^4^(21) ring motifs to form a three-dimensional network.

## Related literature

For general background, see: Aoki *et al.* (1973 ▶, 1974 ▶); Tsi & Tan (1997 ▶); Roy & Sarkar (2005 ▶); Bellasio (1974 ▶, 1975 ▶). For related structures, see: Büyükgüngör & Odabaşoğlu (2006 ▶); Odabaşoğlu & Büyükgüngör (2006 ▶). For ring motif details, see: Bernstein *et al.* (1995 ▶); Etter (1990 ▶).
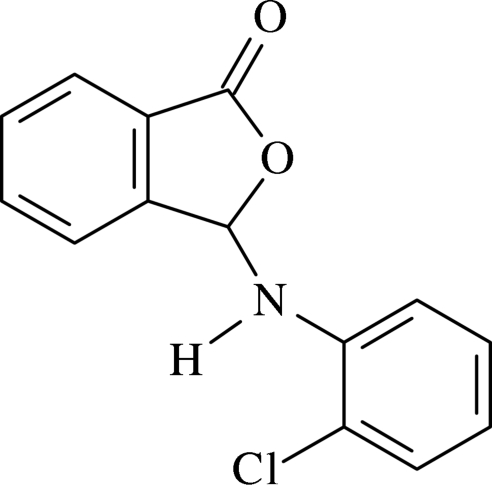


## Experimental

### 

#### Crystal data

C_14_H_10_ClNO_2_
*M* *_r_* = 259.68
Monoclinic, 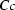

*a* = 9.2485 (8) Å
*b* = 22.7915 (13) Å
*c* = 7.1111 (6) Å
β = 123.823 (6)°
*V* = 1245.25 (19) Å^3^
*Z* = 4
Mo *K*α radiation
μ = 0.30 mm^−1^
*T* = 296 K
0.51 × 0.34 × 0.11 mm

#### Data collection

Stoe IPDSII diffractometer
Absorption correction: integration (*X-RED32*; Stoe & Cie, 2002 ▶) *T*_min_ = 0.880, *T*_max_ = 0.969
7423 measured reflections
2433 independent reflections
2200 reflections with *I* > 2σ(*I*)
*R*_int_ = 0.037

#### Refinement

*R*[*F* ^2^ > 2σ(*F* ^2^)] = 0.029
*wR*(*F* ^2^) = 0.067
*S* = 1.05
2433 reflections
168 parameters
2 restraints
H atoms treated by a mixture of independent and constrained refinement
Δρ_max_ = 0.12 e Å^−3^
Δρ_min_ = −0.15 e Å^−3^
Absolute structure: Flack (1983 ▶), 1205 Friedel pairs
Flack parameter: 0.01 (5)

### 

Data collection: *X-AREA* (Stoe & Cie, 2002 ▶); cell refinement: *X-AREA*; data reduction: *X-RED32* (Stoe & Cie, 2002 ▶); program(s) used to solve structure: *SHELXS97* (Sheldrick, 2008 ▶); program(s) used to refine structure: *SHELXL97* (Sheldrick, 2008 ▶); molecular graphics: *ORTEP-3 for Windows* (Farrugia, 1997 ▶); software used to prepare material for publication: *WinGX* (Farrugia, 1999 ▶).

## Supplementary Material

Crystal structure: contains datablocks I. DOI: 10.1107/S160053680800771X/hk2437sup1.cif

Structure factors: contains datablocks I. DOI: 10.1107/S160053680800771X/hk2437Isup2.hkl

Additional supplementary materials:  crystallographic information; 3D view; checkCIF report

## Figures and Tables

**Table 1:** Hydrogen-bond geometry (Å, °)

*D*—H⋯*A*	*D*—H	H⋯*A*	*D*⋯*A*	*D*—H⋯*A*
N1—H1⋯O1^i^	0.84 (3)	2.29 (3)	3.091 (2)	159 (2)
C4—H4⋯O2^ii^	0.93	2.54	3.397 (2)	153

Symmetry codes: (i) 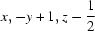; (ii) 
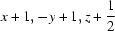
.

**Fig. 1.**
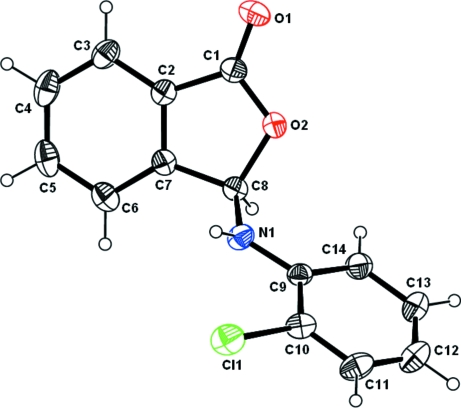
The molecular structure of the title molecule, with the atom-numbering scheme. Displacement ellipsoids are drawn at the 30% probability level.

**Fig. 2.**
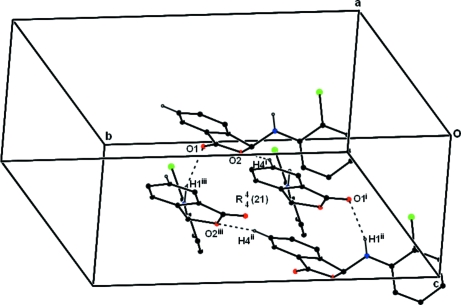
A partial packing diagram of (I), showing the formation of R44(21) ring motif. Hydrogen bonds are shown as dashed lines [symmetry codes: (i) x - 1/2, 1 - y, z - 1/2; (ii) x - 1, y, z; (iii) x, 1 - y, 1/2 + z]. H atoms not involved in hydrogen bondings have been omitted for clarity.

## supplementary crystallographic information

### Comment

Phthalides (isobenzofuranones) are five-membered lactones found in plants and they are known to show diverse biological activities, such as fungicidal, bactericidal, herbicidal, analgesic, pesticidal, hypotensive and vasorelaxant activities (Aoki *et al.*,  1973; Tsi & Tan, 1997; Roy & Sarkar, 2005). In addition, phthalide derivatives are useful in the treatment of circulatory and heart-related diseases (Bellasio, 1974). As part of our ongoing research on 3-substituted phthalides, the title compound, (I), has been synthesized and its crystal structure is reported here.

In the molecule of (I), (Fig. 1), rings A (C2-C7), B (C1/C2/C7/C8/O2) and C (C9-C14) are, of course, planar. The dihedral angles between them are A/B = 2.45 (4)°, A/C = 59.93 (4)° and B/C = 58.90 (4)°. So, rings A and B are also nearly coplanar. Ring C is oriented with respect to the coplanar ring system at a dihedral angle of 59.43 (4)°. The geometry of (I) does not show any significant difference from the average geometry found for 3-(4-chloroanilino)isobenzofuran-1(3*H*)-one (Büyükgüngör & Odabaşoğlu, 2006).

In the crystal structure, intermolecular C-H···O and N-H···O hydrogen bonds (Table 1) link the molecules, generating R_4_^4^(21) (Fig. 2) ring motifs (Bernstein *et al.*,  1995; Etter, 1990), to form a three-dimensional network, in which they may be effective in the stabilization of the structure.

### Experimental

The title compound was prepared according to the method described by Odabaşoğlu & Büyükgüngör (2006), using phthalaldehydic acid and 2-chloroaniline as starting materials (yield; 84%). Crystals of (I) suitable for X-ray analysis were obtained by slow evaporation of an ethanol-DMF (1:1) solution at room temperature.

### Refinement

H atom (for NH) was located in difference synthesis and refined freely [N-H = 0.84 (3) Å and U_iso_(H) = 0.061 (6) Å^2^]. The remaining H atoms were positioned geometrically, with C-H = 0.93 and 0.98 Å for aromatic and methine H, respectively, and constrained to ride on their parent atoms with U_iso_(H) = 1.2U_eq_(C).

### Crystal data

**Table:**

C_14_H_10_ClNO_2_	*F*_000_ = 536
*M**_r_* = 259.68	*D*_x_ = 1.385 Mg m^−^^3^
Monoclinic, *C**c*	Mo *K*α radiation λ = 0.71073 Å
Hall symbol: C -2yc	Cell parameters from 7423 reflections
*a* = 9.2485 (8) Å	θ = 1.8–28.0º
*b* = 22.7915 (13) Å	µ = 0.30 mm^−^^1^
*c* = 7.1111 (6) Å	*T* = 296 K
β = 123.823 (6)º	Prism, colorless
*V* = 1245.25 (19) Å^3^	0.51 × 0.34 × 0.11 mm
*Z* = 4	

### Data collection

**Table:**

Stoe IPDS II diffractometer	2433 independent reflections
Monochromator: plane graphite	2200 reflections with *I* > 2σ(*I*)
Detector resolution: 6.67 pixels mm^-1^	*R*_int_ = 0.038
*T* = 296 K	θ_max_ = 26.0º
w–scan rotation method	θ_min_ = 1.8º
Absorption correction: integration(X-RED32; Stoe & Cie, 2002)	*h* = −11→11
*T*_min_ = 0.880, *T*_max_ = 0.969	*k* = −28→28
7423 measured reflections	*l* = −8→8

### Refinement

**Table:**

Refinement on *F*^2^	Hydrogen site location: inferred from neighbouring sites
Least-squares matrix: full	H atoms treated by a mixture of independent and constrained refinement
*R*[*F*^2^ > 2σ(*F*^2^)] = 0.029	*w* = 1/[σ^2^(*F*_o_^2^) + (0.0412*P*)^2^] where *P* = (*F*_o_^2^ + 2*F*_c_^2^)/3
*wR*(*F*^2^) = 0.067	(Δ/σ)_max_ < 0.001
*S* = 1.05	Δρ_max_ = 0.12 e Å^−^^3^
2433 reflections	Δρ_min_ = −0.15 e Å^−^^3^
168 parameters	Extinction correction: none
2 restraints	Absolute structure: Flack (1983), with 1205 Friedel pairs
Primary atom site location: structure-invariant direct methods	Flack parameter: 0.01 (5)
Secondary atom site location: difference Fourier map	

### Special details

**Table:**

Geometry. All e.s.d.'s (except the e.s.d. in the dihedral angle between two l.s. planes) are estimated using the full covariance matrix. The cell e.s.d.'s are taken into account individually in the estimation of e.s.d.'s in distances, angles and torsion angles; correlations between e.s.d.'s in cell parameters are only used when they are defined by crystal symmetry. An approximate (isotropic) treatment of cell e.s.d.'s is used for estimating e.s.d.'s involving l.s. planes.
Refinement. Refinement of F^2^ against ALL reflections. The weighted R-factor wR and goodness of fit S are based on F^2^, conventional R-factors R are based on F, with F set to zero for negative F^2^. The threshold expression of F^2^ > 2sigma(F^2^) is used only for calculating R-factors(gt) etc. and is not relevant to the choice of reflections for refinement. R-factors based on F^2^ are statistically about twice as large as those based on F, and R- factors based on ALL data will be even larger.

### Fractional atomic coordinates and isotropic or equivalent isotropic displacement parameters (Å^2^)

**Table:**

	*x*	*y*	*z*	*U*_iso_*/*U*_eq_	
Cl1	0.32584 (7)	0.29431 (2)	−0.03793 (8)	0.06502 (16)	
O1	0.48884 (19)	0.56863 (6)	0.4723 (2)	0.0568 (4)	
O2	0.43774 (16)	0.47290 (5)	0.4752 (2)	0.0462 (3)	
N1	0.4488 (2)	0.37749 (6)	0.3398 (3)	0.0446 (3)	
H1	0.462 (3)	0.3829 (10)	0.233 (5)	0.061 (6)*	
C1	0.5400 (2)	0.51868 (7)	0.5030 (3)	0.0418 (4)	
C2	0.7123 (2)	0.49617 (8)	0.5728 (3)	0.0401 (4)	
C3	0.8602 (3)	0.52646 (9)	0.6291 (3)	0.0507 (4)	
H3	0.8604	0.5672	0.6209	0.061*	
C4	1.0076 (3)	0.49429 (11)	0.6977 (3)	0.0616 (5)	
H4	1.1095	0.5134	0.7368	0.074*	
C5	1.0055 (3)	0.43377 (11)	0.7092 (4)	0.0622 (6)	
H5	1.1068	0.4129	0.7568	0.075*	
C6	0.8566 (3)	0.40333 (9)	0.6516 (3)	0.0545 (5)	
H6	0.8558	0.3626	0.6584	0.065*	
C7	0.7097 (2)	0.43588 (7)	0.5838 (3)	0.0404 (4)	
C8	0.5336 (2)	0.41624 (7)	0.5234 (3)	0.0400 (3)	
H8	0.5456	0.3977	0.6558	0.048*	
C9	0.2979 (2)	0.34797 (7)	0.2808 (3)	0.0385 (4)	
C10	0.2263 (3)	0.30630 (8)	0.1070 (3)	0.0473 (4)	
C11	0.0825 (3)	0.27393 (9)	0.0501 (3)	0.0610 (6)	
H11	0.0388	0.2462	−0.0651	0.073*	
C12	0.0027 (3)	0.28242 (11)	0.1632 (4)	0.0678 (6)	
H12	−0.0959	0.2610	0.1235	0.081*	
C13	0.0701 (3)	0.32296 (9)	0.3359 (4)	0.0588 (5)	
H13	0.0174	0.3285	0.4141	0.071*	
C14	0.2156 (2)	0.35546 (8)	0.3937 (3)	0.0488 (4)	
H14	0.2592	0.3828	0.5101	0.059*	

### Atomic displacement parameters (Å^2^)

**Table:**

	*U*^11^	*U*^22^	*U*^33^	*U*^12^	*U*^13^	*U*^23^
Cl1	0.0858 (4)	0.0647 (3)	0.0552 (2)	−0.0121 (3)	0.0459 (3)	−0.0150 (2)
O1	0.0704 (10)	0.0411 (7)	0.0633 (8)	0.0097 (6)	0.0399 (8)	0.0004 (6)
O2	0.0393 (6)	0.0438 (7)	0.0576 (7)	0.0011 (5)	0.0282 (6)	−0.0031 (5)
N1	0.0508 (9)	0.0446 (8)	0.0439 (8)	−0.0085 (7)	0.0297 (7)	−0.0057 (6)
C1	0.0468 (10)	0.0416 (9)	0.0371 (8)	0.0008 (8)	0.0234 (7)	−0.0028 (7)
C2	0.0406 (9)	0.0446 (9)	0.0332 (8)	−0.0032 (7)	0.0192 (7)	−0.0032 (6)
C3	0.0494 (11)	0.0577 (11)	0.0431 (8)	−0.0141 (9)	0.0245 (8)	−0.0071 (8)
C4	0.0403 (11)	0.0914 (16)	0.0487 (11)	−0.0170 (10)	0.0221 (9)	−0.0092 (10)
C5	0.0379 (10)	0.0905 (17)	0.0527 (11)	0.0122 (10)	0.0218 (9)	0.0000 (10)
C6	0.0496 (11)	0.0555 (11)	0.0552 (10)	0.0111 (9)	0.0271 (9)	0.0032 (9)
C7	0.0379 (9)	0.0445 (9)	0.0363 (8)	0.0015 (7)	0.0192 (7)	−0.0013 (7)
C8	0.0408 (9)	0.0381 (8)	0.0404 (8)	0.0015 (7)	0.0220 (7)	0.0001 (7)
C9	0.0399 (9)	0.0313 (8)	0.0405 (8)	0.0008 (7)	0.0201 (7)	0.0033 (6)
C10	0.0552 (11)	0.0431 (9)	0.0378 (8)	−0.0032 (8)	0.0223 (8)	−0.0001 (7)
C11	0.0629 (14)	0.0537 (11)	0.0523 (11)	−0.0203 (10)	0.0233 (10)	−0.0118 (9)
C12	0.0591 (13)	0.0671 (14)	0.0748 (14)	−0.0228 (10)	0.0358 (12)	−0.0053 (10)
C13	0.0551 (12)	0.0593 (13)	0.0726 (13)	−0.0092 (10)	0.0422 (11)	−0.0001 (10)
C14	0.0535 (11)	0.0456 (10)	0.0522 (10)	−0.0025 (8)	0.0324 (9)	−0.0022 (7)

### Geometric parameters (Å, °)

**Table:**

N1—H1	0.84 (3)	C8—N1	1.400 (2)
C1—O1	1.205 (2)	C8—O2	1.494 (2)
C1—O2	1.347 (2)	C8—H8	0.9800
C1—C2	1.472 (3)	C9—N1	1.387 (2)
C2—C7	1.377 (2)	C9—C14	1.391 (2)
C2—C3	1.378 (3)	C9—C10	1.399 (2)
C3—C4	1.376 (3)	C10—C11	1.370 (3)
C3—H3	0.9300	C10—Cl1	1.744 (2)
C4—C5	1.383 (4)	C11—C12	1.375 (3)
C4—H4	0.9300	C11—H11	0.9300
C5—C6	1.385 (3)	C12—C13	1.377 (3)
C5—H5	0.9300	C12—H12	0.9300
C6—C7	1.378 (3)	C13—C14	1.383 (3)
C6—H6	0.9300	C13—H13	0.9300
C7—C8	1.503 (3)	C14—H14	0.9300
			
C1—O2—C8	110.92 (13)	N1—C8—O2	112.25 (14)
C8—N1—H1	117.4 (16)	N1—C8—C7	114.17 (15)
C9—N1—C8	122.34 (16)	O2—C8—C7	102.62 (13)
C9—N1—H1	115.2 (17)	N1—C8—H8	109.2
O1—C1—O2	122.15 (17)	O2—C8—H8	109.2
O1—C1—C2	129.22 (17)	C7—C8—H8	109.2
O2—C1—C2	108.63 (14)	N1—C9—C14	123.22 (15)
C7—C2—C3	121.94 (17)	N1—C9—C10	119.92 (15)
C7—C2—C1	108.50 (15)	C14—C9—C10	116.80 (16)
C3—C2—C1	129.53 (17)	C11—C10—C9	122.08 (18)
C4—C3—C2	117.61 (19)	C11—C10—Cl1	119.09 (14)
C4—C3—H3	121.2	C9—C10—Cl1	118.82 (14)
C2—C3—H3	121.2	C10—C11—C12	120.02 (19)
C3—C4—C5	120.61 (19)	C10—C11—H11	120.0
C3—C4—H4	119.7	C12—C11—H11	120.0
C5—C4—H4	119.7	C11—C12—C13	119.48 (19)
C4—C5—C6	121.76 (19)	C11—C12—H12	120.3
C4—C5—H5	119.1	C13—C12—H12	120.3
C6—C5—H5	119.1	C12—C13—C14	120.4 (2)
C7—C6—C5	117.26 (19)	C12—C13—H13	119.8
C7—C6—H6	121.4	C14—C13—H13	119.8
C5—C6—H6	121.4	C13—C14—C9	121.18 (17)
C2—C7—C6	120.82 (17)	C13—C14—H14	119.4
C2—C7—C8	109.32 (14)	C9—C14—H14	119.4
C6—C7—C8	129.80 (16)		
			
O1—C1—O2—C8	179.77 (16)	C2—C7—C8—O2	0.52 (17)
C2—C1—O2—C8	−0.24 (16)	C6—C7—C8—O2	177.69 (17)
O1—C1—C2—C7	−179.43 (17)	N1—C8—O2—C1	−123.18 (15)
O2—C1—C2—C7	0.59 (17)	C7—C8—O2—C1	−0.16 (16)
O1—C1—C2—C3	2.8 (3)	O2—C8—N1—C9	−73.5 (2)
O2—C1—C2—C3	−177.14 (15)	C7—C8—N1—C9	170.20 (15)
C7—C2—C3—C4	0.0 (3)	C14—C9—N1—C8	1.9 (3)
C1—C2—C3—C4	177.43 (17)	C10—C9—N1—C8	−174.97 (15)
C2—C3—C4—C5	0.0 (3)	N1—C9—C10—C11	176.68 (18)
C3—C4—C5—C6	0.3 (3)	C14—C9—C10—C11	−0.3 (2)
C4—C5—C6—C7	−0.5 (3)	N1—C9—C10—Cl1	−2.1 (2)
C3—C2—C7—C6	−0.2 (3)	C14—C9—C10—Cl1	−179.13 (13)
C1—C2—C7—C6	−178.15 (15)	C9—C10—C11—C12	0.8 (3)
C3—C2—C7—C8	177.25 (14)	Cl1—C10—C11—C12	179.56 (18)
C1—C2—C7—C8	−0.68 (18)	C10—C11—C12—C13	−1.0 (4)
C5—C6—C7—C2	0.5 (3)	C11—C12—C13—C14	0.8 (4)
C5—C6—C7—C8	−176.40 (17)	C12—C13—C14—C9	−0.3 (3)
C2—C7—C8—N1	122.25 (16)	N1—C9—C14—C13	−176.81 (18)
C6—C7—C8—N1	−60.6 (2)	C10—C9—C14—C13	0.1 (3)

### Hydrogen-bond geometry (Å, °)

**Table:**

*D*—H···*A*	*D*—H	H···*A*	*D*···*A*	*D*—H···*A*
N1—H1···O1^i^	0.84 (3)	2.29 (3)	3.091 (2)	159 (2)
C4—H4···O2^ii^	0.93	2.54	3.397 (2)	153

Symmetry codes: (i) *x*, −*y*+1, *z*−1/2; (ii) *x*+1, −*y*+1, *z*+1/2.
